# Endoscopic submucosal dissection for hypopharynx lymphoepithelioma-like carcinoma

**DOI:** 10.1055/a-2233-3082

**Published:** 2024-01-23

**Authors:** Cheng Guo, Bensong Duan, Li Zhang, Meidong Xu, Haibin Zhang

**Affiliations:** 1 66324 Endoscopy Center, Department of Gastroenterology, Shanghai East Hospital, Shanghai, China; 2 66324 Department of Pathology, Shanghai East Hospital, Shanghai, China

A 48-year-old man with no significant medical history presented to our hospital with a 1-year history of a foreign body sensation in the pharynx. Upper endoscopy revealed a mass originating from the left aryepiglottic fold, which completely filled the pyriform sinus and partially obstructed the entrance to the esophagus ( Fig. 1 **a** ). Ultrasound endoscopy revealed a well-demarcated hypoechoic mass with uniform echogenicity, which was chiefly located in the left aryepiglottic fold ( Fig. 1 **b** ). With a diagnosis of giant inflammatory fibroma suspected, endoscopic submucosal dissection (ESD) was performed to prevent incarcerated obstruction ( Fig. 1 **c,d** , Video 1 ). Histological results confirmed the mass to be lymphoepithelioma-like carcinoma (LELC) ( Fig. 2 ). The patient subsequently attended for follow-up endoscopy after a year, during which no evidence of recurrence or complications such as stenosis was seen.

**Fig. 1:**
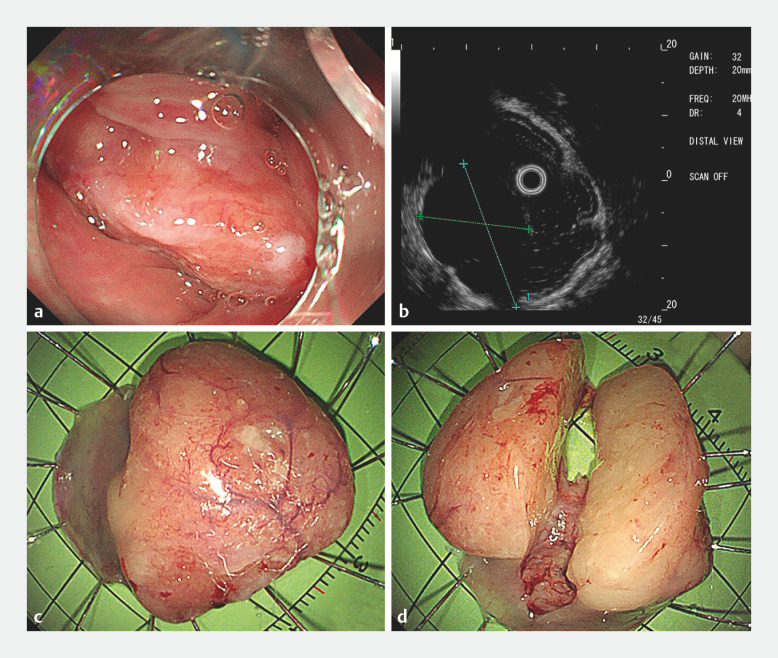
The mass originated from the left aryepiglottic fold and was removed endoscopically. **a** The mass filled the pyriform sinus and obstructed the entrance to the esophagus. **b** Ultrasound endoscopy observation. **c,d** The whole specimen was 3.0×2.5×2.5 cm.

Endoscopic submucosal dissection was performed to remove a mass in the hypopharynx.Video 1

**Fig. 2:**
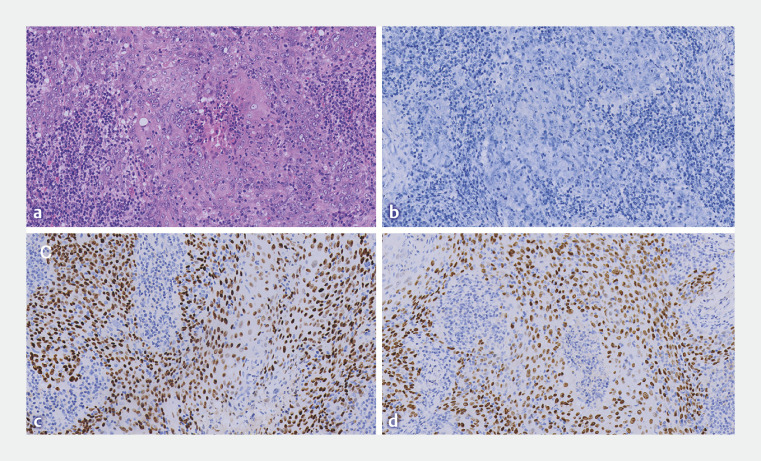
Hematoxylin and eosin (HE) and immunohistochemistry analysis. **a** HE revealed nests of neoplastic epithelial cells separated by abundant lymphoid stroma. **b** The specimen was negative for Epstein–Barr virus-encoded RNAs (EBERs). **c,d** The specimen was positive for P40 ( **c** ) and P63 ( **d** ).

LELC is a rare histological malignancy type characterized by the presence of a markedly prominent lymphoid infiltration and commonly associated with Epstein–Barr virus (EBV) infection 1 . Interestingly, this patient did not have EBV infection. Surgery and chemoradiotherapy are optional treatments but may lead to swallowing difficulties, vocal disturbances, and other side effects. ESD is commonly performed for early gastrointestinal cancers. However, pharyngeal ESD is still a challenge for most endoscopists owing to the limited working space and inexperience. Iizuka et al. reported the advantages of ESD in pharyngeal squamous cell carcinoma 2 . Our previous study reported the effectiveness of ESD for superficial pharyngeal carcinoma 3 . The current case is the first report of a hypopharynx LELC that was completely resected by endoscopy, and demonstrates the potential of ESD.

Endoscopy_UCTN_Code_TTT_1AO_2AG

## Notes

Endoscopy E-Videos https://eref.thieme.de/e-videos
*E-Videos* is an open access online section of the journal *Endoscopy* , reporting on interesting cases and new techniques in gastroenterological endoscopy. All papers include a high-quality video and are published with a Creative Commons CC-BY license. Endoscopy E-Videos qualify for HINARI discounts and waivers and eligibility is automatically checked during the submission process. We grant 100% waivers to articles whose corresponding authors are based in Group A countries and 50% waivers to those who are based in Group B countries as classified by Research4Life (see: https://www.research4life.org/access/eligibility/ ). This section has its own submission website at https://mc.manuscriptcentral.com/e-videos .
